# MetaFX: feature extraction from whole-genome metagenomic sequencing data

**DOI:** 10.1093/bioinformatics/btag018

**Published:** 2026-01-20

**Authors:** Artem Ivanov, Vladimir Popov, Maxim Morozov, Evgenii Olekhnovich, Vladimir Ulyantsev

**Affiliations:** Information Technology and Programming Department, ITMO University, St. Petersburg 197101, Russia; Genome Research and Computational Biology Lab, Lopukhin Federal Research and Clinical Center of Physical-Chemical Medicine of Federal Medical Biological Agency, Moscow 119435, Russia; School of Mathematics and Mechanics, St. Petersburg State University, St. Petersburg 199034, Russia; Genome Research and Computational Biology Lab, Lopukhin Federal Research and Clinical Center of Physical-Chemical Medicine of Federal Medical Biological Agency, Moscow 119435, Russia; Genome Research and Computational Biology Lab, Lopukhin Federal Research and Clinical Center of Physical-Chemical Medicine of Federal Medical Biological Agency, Moscow 119435, Russia; Information Technology and Programming Department, ITMO University, St. Petersburg 197101, Russia

## Abstract

**Motivation:**

Microbial communities consist of thousands of microorganisms and viruses and have a tight connection with an environment, such as gut microbiota modulation of host body metabolism. However, the direct relationship between the presence of certain microorganism and the host state often remains unknown. Toolkits using reference-based approaches are limited to microbes present in databases. Reference-free methods often require enormous resources for metagenomic assembly or results in many poorly interpretable features based on k-mers.

**Results:**

Here we present MetaFX—an open-source library for feature extraction from whole-genome metagenomic sequencing data and classification of groups of samples. Using a large volume of metagenomic samples deposited in databases, MetaFX compares samples grouped by metadata criteria (e.g. disease, treatment, etc.) and constructs genomic features distinct for certain types of communities. Features constructed based on statistical k-mer analysis and de Bruijn graphs partition. Those features are used in machine learning models for classification of novel samples. Extracted features can be visualized on de Bruijn graphs and annotated for providing biological insights. We demonstrate the utility of MetaFX by building classification models for 590 human gut samples with inflammatory bowel disease. Our results outperform the previous research disease prediction accuracy up to 17%, and improves classification results compared to taxonomic analysis by 9±10% on average.

**Availability and implementation:**

MetaFX is a feature extraction toolkit applicable for metagenomic datasets analysis and samples classification. The source code, test data, and relevant information for MetaFX are freely accessible at https://github.com/ctlab/metafx under the MIT License. Alternatively, MetaFX can be obtained via http://doi.org/10.5281/zenodo.16949369.

## 1 Introduction

Microbial communities inhabit diverse ecological niches, including soil, water reservoirs, the human gut, and others (Human Microbiome Project Consortium 2012, Fierer 2017, Garner *et al.* 2023). Metagenomic sequencing data analysis is a well-established approach to analyse the structure and functions of microbial communities. For example, many ongoing studies are exploring the gut microbiome with regards to health and disease, as well as contributing to the development of diagnostic and therapeutic strategies (Qin *et al.* 2012, Jie *et al.* 2017, Yu *et al.* 2017, Lloyd-Price *et al.* 2019, Ivanova *et al.* 2022, Olekhnovich *et al.* 2021, Olekhnovich *et al.* 2023). However, existing metagenomic analysis methods, including taxonomic and functional anotation or genome-resolved metagenomics, provide only limited resolution of microbiome properties coupled with the limited completeness of reference databases. At present, two paradigms of metagenomic data analysis can be distinguished: reference-based and reference free.

Reference-based methods include a wide range of applications for analysing metagenomes using reference sequences to perform taxonomic and functional annotation (Beghini *et al.* 2021, Lu *et al.* 2022, Blanco-Míguez *et al.* 2023, Shaw and Yu 2024). Reference sequences may consist of gene sequences, metagenome-assembled genomes (MAGs), whole genomes, or parts of them. Overall, this type of analysis is limited by the completeness of databases and does not allow information on “microbial dark matter” to be included in the analysis. This may result in the loss of a crucial biological relation, which might be essential for machine learning models, among other things. Though, the addition of “lost” data may improve the efficacy of these models and contribute to a deeper understanding of the underlying biology of the issues in question.

Another group of methods for metagenomic analysis is reference-free methods. Initially, these applications included genome-resolved metagenomic techniques. Discovering uncultivated microbial or viral diversity may be facilitated by recovering diversity directly from metagenomic data utilizing genomes assembled from metagenomes. However, such pipelines are not always relevant when the researcher is faced with the task of quickly extracting information from metagenomes. Moreover, some of the useful information is also lost in the process of metagenome assembly and filtering out low-quality bins. Thus, there is a need to create algorithms that can process large datasets quickly but still work directly with the data without loss of information.

K-mer based approaches are alternative ways to analyse metagenomic data. They do not provide an in-depth understanding of microbial community structure, but allow for rapid exploratory data analysis to generate initial hypotheses. An additional advantage of this method is the inclusion of the unannotated part of the metagenome containing “microbial or viral dark matter.” These methods include Mash (Ondov *et al.* 2016) and MetaFast (Ulyantsev *et al.* 2016) for rapid metagenome comparison, MetaCherchant (Olekhnovich *et al.* 2018) for extracting the metagenomic environment of the target gene of interest, and RECAST (Olekhnovich *et al.* 2021) for comparing donor and patient metagenomic sets in faecal transplantation experiments. The k-mer-based feature selection methods include approaches such as Jellyfish (Marçais and Kingsford 2011), Commet (Maillet *et al.* 2014), Simka (Benoit *et al.* 2016), KMC3 (Kokot *et al.* 2017), KmerGO (Wang *et al.* 2020). Nevertheless, they do not allow further analysis of the identified features using taxonomic and functional annotation methods which makes biological interpretation of the results difficult.

Here, we present MetaFX, an open-source library for the extraction of features from metagenomic sequencing data and the classification of samples based on these features. Its main aim is to construct features initially in a reference-free manner, while keeping the possibility for their subsequent analysis and annotation. The tool takes as an input metagenomic data in fastq or fasta format from short-read Illumina sequencing platforms and constructs features in the form of contigs or branching paths in the de Bruijn graph. These features can be further analysed for biological significance (e.g. taxonomic or functionally annotated) as well as be used to train machine learning models for classification of novel samples.

## 2 Materials and Methods

### 2.1 Concept of MetaFX

**Figure 1:**
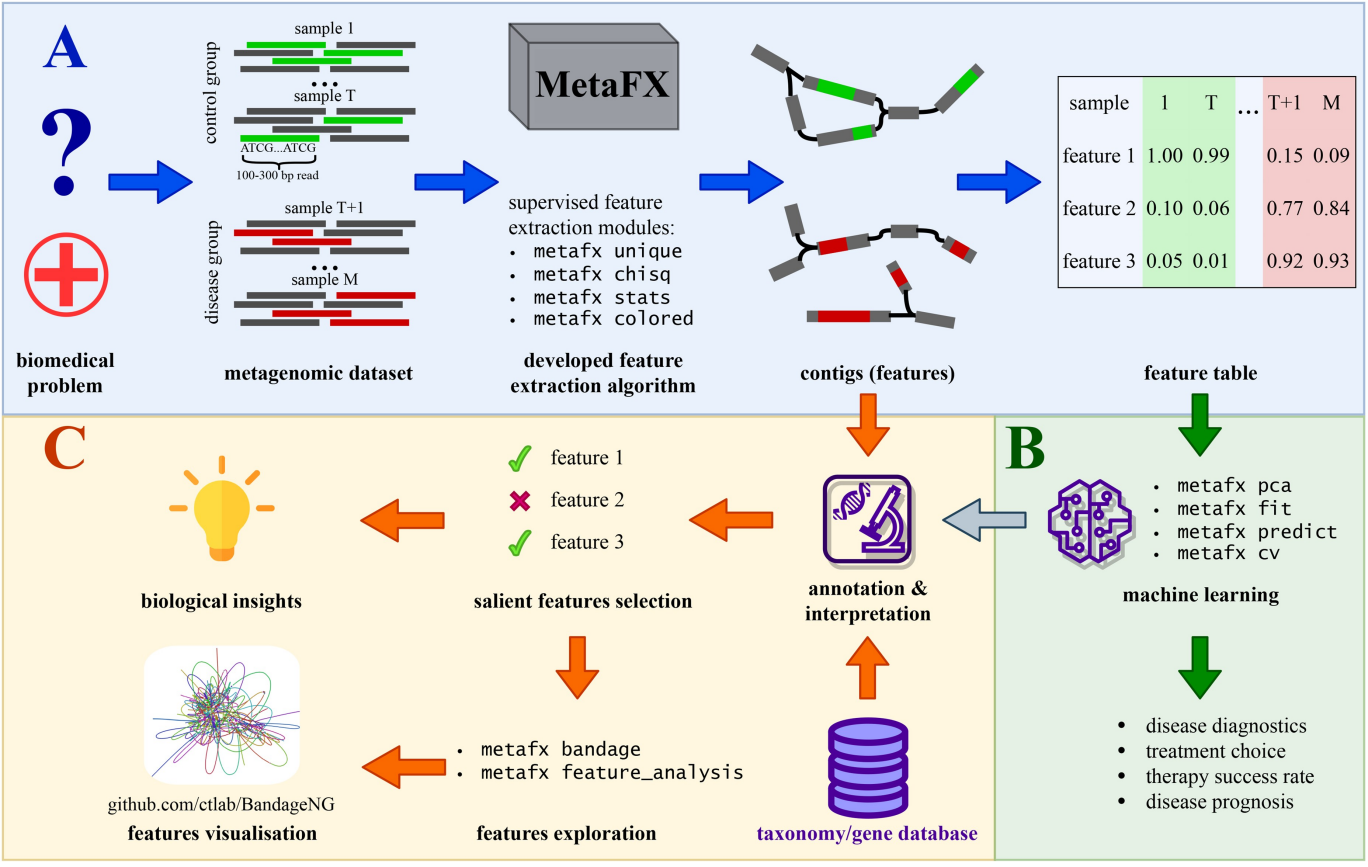
Overall pipeline of data analysis from raw reads to interpretable features and classification model in MetaFX. (A) Feature extraction steps: raw sequencing reads are processed using k-mers and de Bruijn graph approaches to obtain long nucleotide sequences and numeric feature table; (B) machine learning models training steps, including dimensionality reduction and Random Forest classification; (C) features annotation with taxonomy and functional databases and visualization of features and trained classifier.

MetaFX is a command line tool optimized for multi-threaded environments, which makes it possible to process large metagenomic datasets (hundreds of samples) on computational servers within a reasonable time limit (e.g. 220 samples were successfully processed in 40 h with 32 threads and 400 Gb RAM). The outline of MetaFX library is presented in Fig. 1. MetaFX is implemented as a pipeline, which calls several tools for feature extraction and visualizations, and custom python scripts for data analysis and manipulation. Most of the feature extraction modules are based on the MetaFast (Ulyantsev *et al.* 2016) toolkit, which was substantially modified by creating new classes for supervised feature extraction using samples metadata. The idea for MetaFX raised from international challenge on inflammatory bowel disease (IBD) prediction based on patients gut microbiome, that authors of this paper won (Khachatryan *et al.* 2023).

Conceptually, all MetaFX modules can be divided into three categories (Fig. 1, available as supplementary data at *Bioinformatics* online). Two of them aimed at constructing distinct features from samples. Features can be extracted jointly from all of the input metagenomes in case of the lack of associated samples metadata—these methods are described in unsupervised methods section. Alternatively, all samples can be split into several groups based on samples’ metadata information. In that case, supervised methods are applied to extract features for each group independently. The last group of methods encompass various scripts for subsequent analysis of extracted features. It includes classification model training and prediction, preparation for features visualization, and also utilities for computations speed up and formats transformations.

### 2.2 Unsupervised methods for feature extraction

Unsupervised methods provide the ability to extract features for all input samples. It can be useful for exploratory data analysis and samples clustering when there is no *a priori* division of metagenomes into categories. Two such methods have been implemented.

MetaFX metafast module uses original MetaFast algorithm to construct condensed de Bruijn graph of all samples. Then it is iteratively broken into connected components based on coverage thresholds (see details in Ulyantsev *et al.* 2016). Each component then used as a single feature. Users can tune the number of components and their sizes by controlling the input parameters such as coverage thresholds.

Another module metaspades utilizes metaSPAdes assembler (Nurk *et al.* 2017) to extract contigs from each sample independently. It can be useful in the case where assembly is planned to be performed during a study, as the assembly results are saved and can be reused. Next, the resulting contigs are transformed into features either directly or by combining them via de Bruijn graph construction.

Transition from graph components to the numerical features is done via k-mers alignment back to the components and calculation of depth and breadth coverage for each sample independently. The result of the feature extraction step is identical for all samples. For each sample and for each graph component the following steps are performed:

1. Take one component and split it into k-mers.
2. Make an intersection of component’s k-mers set and sample’s k-mers set.
3. Count the size of intersection and divide it by the size of the component. The resulting value from 0 to 1 is the *breadth* coverage.
4. The *depth* coverage is calculated by multiplying each common k-mer by it is abundance in the sample. The resulting sum is divided by the size of the component to get positive real number—mean depth coverage.
5. Repeat the steps 1–4 for all components.

As a result, each sample is represented as two numerical vectors of length equal to the number of extracted graph components. *Breadth* showed better performance in MEDIC competition (Khachatryan *et al.* 2023), so it is used by default in automatic pipeline. However, *depth* is also calculated and saved for possibility to further analyse results using custom pipelines.

Finally, all vectors are joined into the table of shape N_features×M_samples. Since the additional metadata for samples is not exploited during features construction (and generally not available, otherwise supervised algorithms should be preferred), the resulting feature table can be used for samples clustering and patterns detection.

### 2.3 Supervised methods for feature extraction

**Figure 2:**
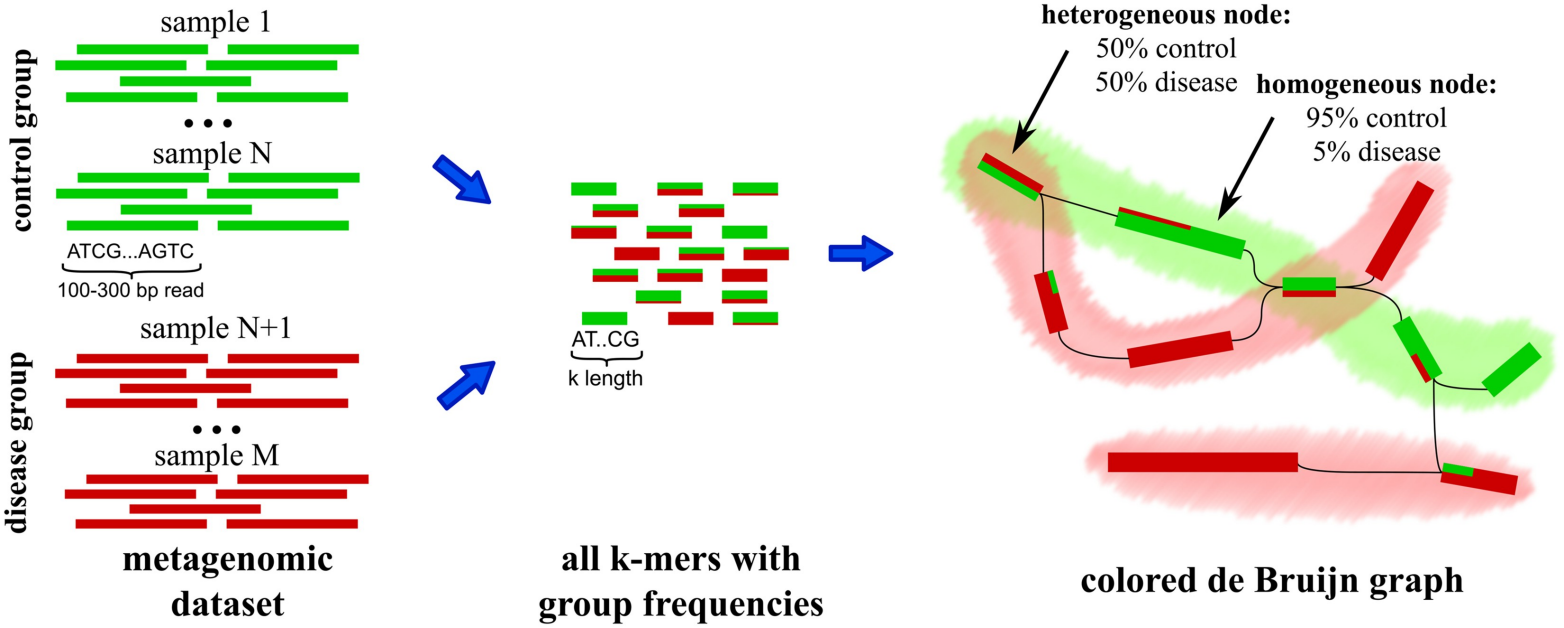
Workflow of coloured feature extraction module. All k-mers are assigned with colours proportionally to the relative occurrence in samples’ groups. Next, de Bruijn graph with coloured vertices is split into components. The extracted features are highlighted with shades (green for control and red for disease group).

Should the input dataset have the associated metadata to split samples into several categories, supervised methods can be applied to extract features specific for each group. Overall scheme of features extraction pipelines is presented in Algorithm 1 and on Fig. 2, available as supplementary data at *Bioinformatics* online. MetaFX contains three independent strategies aimed at selection of specific k-mers for each group of samples, implemented in modules unique, chisq, and stats.

Algorithm 1 Supervised feature extraction pipeline

 **Input:** samples with labeled categories

 **Output:** components in fasta format and feature table

1: samples=LoadSamples()

2: category=LoadSampleNamesByCategory()

3: G=CountCategories()

4: **for**  sample∈samples  **do** 

5:   kmers.append(KmersCounter(sample))

6: **end for** 

7: **for**  i∈(1…G)  **do** 

8:   specific[i]=KmersExtractor(category[i],  category[G∖{i}])[1]

9: **end for** 

10: components=ComponentsExtractor(specific,kmers)

11: **for**  sample∈samples  **do** 

12:   values.append(FeaturesCalculator(components,sample))

13: **end for** 

14: table=JoinVectors(values)

15: features=ComponentsToFasta(components)

16: **return**  (features,table)

Unique algorithm searches for group-specific k-mers, defined as a k-mer present in at least *G* samples of certain group and absent in all other samples. *G* parameter can be chosen by researcher or automatically selected to obtain usable number of features (∼1000–10 000) for each category.

Chisq and stats algorithms apply statistical tests to select group-significant k-mers. First method utilize chi-squared test for selection top significant k-mers, while second method combines chi-squared with Mann–Whitney *U* test to select k-mers with different occurrences between categories. Yates’s correction for continuity and Bonferroni correction for multiple comparisons are used.

All three aforementioned methods result in sets of k-mers specific for each category, that need to be transformed into features. Further, selected k-mers are used as pivots for local de Bruijn graph construction. Each category is processed independently. For a given category all samples are split into k-mers and are used to build de Bruijn graph. Next, selected specific k-mers are marked as starting points for search in the graph. For each starting point we perform the procedure of local search, based on combination of depth-first and breadth-first searches with early-stopping criteria. The number of branches studied during the search is defined by the launch parameter. The branches with no group-specific k-mers are discarded. As a result, numerous selected k-mers are grouped into graph components, reducing the resulting number of features. Calculation of numerical feature vectors for the components performed in the same manner as for the unsupervised methods. Finally, each component is saved into fasta file as non-overlapping contigs for further analysis and annotation.

The last MetaFX module for supervised feature extraction is named colored and can process only two or three groups of samples (Fig. 2). This limitation comes from the implementation details. However, the method is still useful for biomedical applications, e.g. comparing disease versus control group, or control versus treatment 1 versus treatment 2.

The distinctive advantage of the colored module is the control of the desired number of resulting features. Unlike the methods described above, it first assigns to each k-mer the probability vector to belong to each of the groups. The length of the vector equals to the number of categories. The value in vector is a ratio of number of samples of given category *G* containing the given k-mer to the total number of samples with that k-mer [see Equation (1)].


(1) $$V_{G}(k-\text{mer})=\frac{\sum_{S\in G}k-\text{mer}\in S}{\sum_{S}k-\text{mer}\in S},\;\text{where}\;\sum_{G}V_{G}(k-\text{mer})=1.$$

Further the global de Bruijn graph for all samples is constructed with nodes assigned colors based on k-mers probabilities. Finally, the coloured graph is split into components. User can select to construct limited number of components for each group, starting from the group most probable k-mers, or to output all components around coloured k-mers with probability above the threshold.1

### 2.4 Building predictive classification models

Having constructed the features, the next step is to analyse them. One option is to use the obtained feature table in machine learning algorithms. For that purpose several python scripts were developed. In case of the unsupervised feature extraction, MetaFX pca algorithm can be used to perform Principal Component Analysis and visualization of samples proximity. The implementation is based on scikit-learn library (Pedregosa *et al.* 2011).

In case of the supervised feature extraction, fit or cv modules are used to train classification model and, optionally, tune the hyper-parameters. Tree-based models remain state-of-the-art classifiers for tabular data analysis (Grinsztajn *et al.* 2022). Consequently, MetaFX supports Random Forest Classifier from scikit-learn library, XGBoost library for building tree-based gradient boosting models (Chen and Guestrin 2016), and PyTorch library with custom neural network to showcase the possible analysis (Paszke *et al.* 2019). However, the resulting feature table can be examined by any method of the researcher’s choice.

The constructed features and pre-trained classifiers are extremely valuable to speed up further research. Suppose the case of new study group of samples with unknown categories from the same type of the environment. Metafx calc_features method should be called to count numeric feature values for new samples based on previously extracted graph features. This allows the researcher to skip the most resource-consuming steps of graph construction and feature extraction. Instead, basic k-mers manipulations enough to obtain numeric features for classification. After that, predict method can be used to classify novel samples with pre-trained model.

### 2.5 Methods for data analysis and visualization

**Figure 3:**
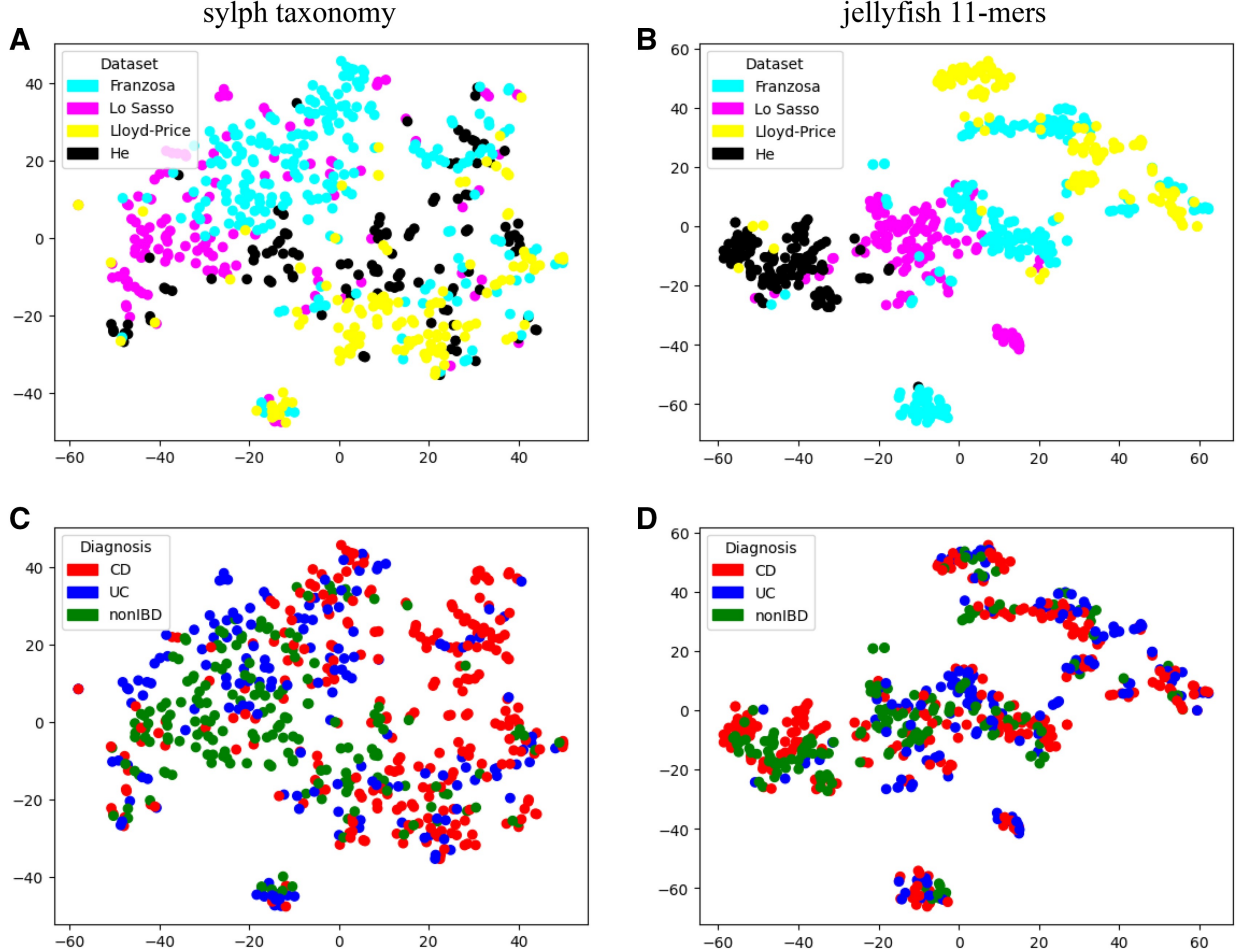
t-SNE visualization of all datasets based on sylph taxonomic annotation (A, C) and on sylph 11-mers (B, D). Colouring modes suggest cohort-specific properties to be generally more pronounced than disease-specific ones.

Another option is to analyse features in-depth and visualize them. For convenient visual representation of extracted features, Bandage program (Wick *et al.* 2015) was substantially improved and the required version is available at https://github.com/ctlab/BandageNG. MetaFX bandage module enables the joint visualization of feature graphs and the trained Random Forest classifier (Fig. 3, available as supplementary data at *Bioinformatics* online). Bandage web-interface is split into two screens: left for de Bruijn graph visualization and right for Random Forest visualization. Nodes in decision trees are annotated with the feature used as criterion. These features can be mapped back to the contigs of de Bruijn graph and highlighted. Thus we can detect the most used features in classification and analyse their graph environment.

Metafx feature_analysis module allows the detailed examination of the selected feature. Feature’s local environment is constructed for each sample via MetaCherchant toolkit (Olekhnovich *et al.* 2018) and then all the environments can be simultaneously visualized in BandageNG. These visualizations can be manually explored for connections not obvious from contigs fasta files.

## 3 Results

MetaFX was tested on real human gut metagenomic dataset of 590 samples (∼1.8 Tb of gzipped reads). Data was collected from 4 studies (He *et al.* 2017, Franzosa *et al.* 2019, Lloyd-Price *et al.* 2019, Lo Sasso *et al.* 2021) focused on gut microbiome analysis of patients with IBD. Samples are grouped into 3 categories: patients with Crohn’s disease (CD), patients with ulcerative colitis (UC) and control group of healthy individuals. Generalized information about datasets is present in Table 1, available as supplementary data at *Bioinformatics* online, per-sample statistics are available from authors’ original articles.

Six strategies were used for feature extraction. As a baseline reference-based approach, we performed taxonomic annotation of all samples via Kraken2 (Wood *et al.* 2019) with *Standard* database of Refseq archaea, bacteria, viral, plasmid, human, and UniVec_Core collected on 14 March 2023 at https://benlangmead.github.io/aws-indexes/k2. Annotations were filtered at species level and relative abundance was used as features in classification models. For better species-level profiling we also performed taxonomic annotation of all samples with sylph (Shaw and Yu 2024) using GTDB r220 database. Taxonomic profiles were obtained via sylph-tax utility using *sequence-abundance* field for consistency with Kraken output.

As a baseline reference-free approach, we performed k-mers extraction from all samples with jellyfish (Marçais and Kingsford 2011). The decision to use k=11 in this method is determined by the large data volume and limited computational resources (i.e. for k=21 we were unable to perform t-SNE analysis nor to train Random Forest model with 800 Gb RAM available). We used only canonical k-mers (-C parameter of Jellyfish).

Three other strategies are implemented in MetaFX. First, reads were processed by splitting them into k-mers of length 31. The choice is inspired by the default *k* value in MetaFast tool as well as default value *l* for minimizers in Kraken2. Each sample was processed independently and all singletons were discarded. As a result, each sample is represented as a set of 31-mers. Next, we applied one unsupervised and two supervised methods for feature extraction.

MetaFX metafast unsupervised algorithm was applied to all samples in all datasets simultaneously, as we pretend the lack of corresponding diagnosis as metadata. MetaFX unique and stats methods were applied to each dataset for samples grouped by diagnosis. As a result of all three methods we obtained feature tables of relative breadth coverage of extracted features by each sample.

Before solving classification problems, we performed exploratory data analysis to better understand samples distribution according to cohort and diagnosis metadata. We applied t-SNE to visualize data points in two-dimensional space. Species-level sylph taxonomic annotation and jellyfish 11-mers were used as features. We ran implementation from scikit-learn package for python with parameters perplexity=10 and Bray-Curtis metric for distance calculation. The results were visualized with matplotlib, showing the samples distributions, coloured by datasets (Fig. 3A and B) and by diagnosis (Fig. 3C,and D). Both image pairs (A & C for taxonomy features and B & D for k-mers features) show better clustering when coloured by dataset rather than by diagnosis. This suggest the cohort-specific properties of the datasets to be present that may complicate the classification and cohort-independent model construction.

**Figure 4:**
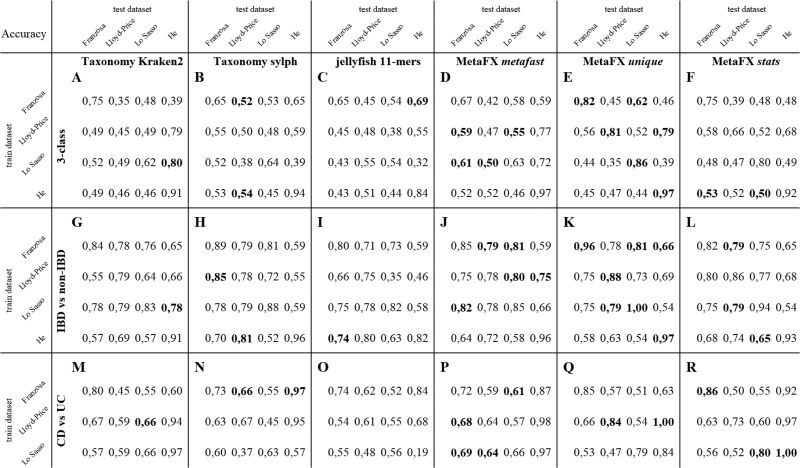
Inflammatory Bowel Disease Random Forest classification accuracy. Three scenarios were analysed: 3-class predictions (A–F), IBD versus non-IBD (G–L), CD versus UC (M–R). For each scenario for each train-test datasets pair the best result is highlighted in bold. 10-fold cross-validation used for the same train-test pair. Unique method is the best inside the cohort. Mostly (in 75% cases) one of the proposed MetaFX methods outperforms others.

We continued investigation by applying Mash tool (Ondov *et al.* 2016) to estimate the distance between metagenomic samples. It was run with sketch size equals 1 000 000 and other settings by default. Based on the obtained distance matrix we performed hierarchical clustering with ward metric from scipy python package. Resulting image (Fig. 4, available as supplementary data at *Bioinformatics* online) does not provide distinct cluster neither for datasets, nor for diagnosis. However, there is a trend for better clustering based on dataset parameter.

Further, we tackled three types of classification problems. First, we aimed to predict one of three classes. Second, we aimed to distinguish between control samples and diseased samples. Finally, we aimed to differentiate between CD and UC diagnosis.

**Figure 5:**
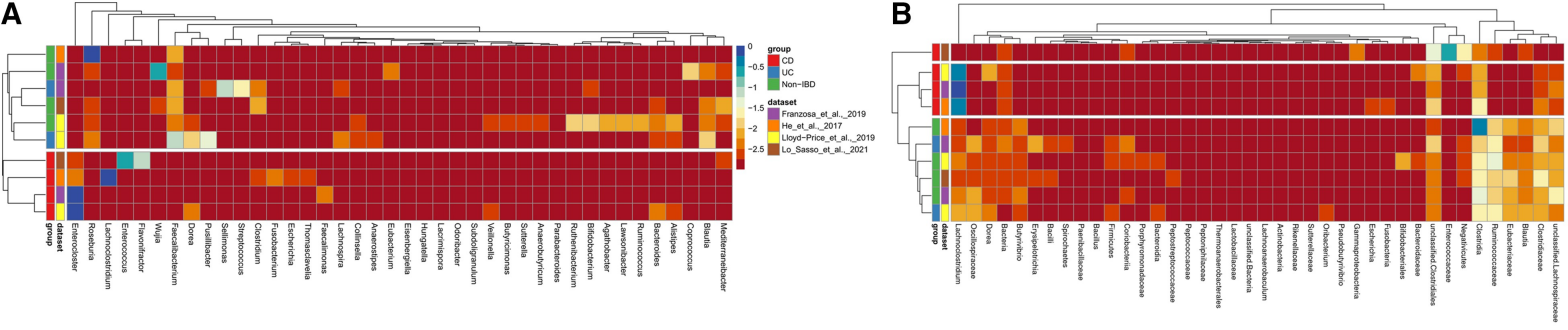
Taxonomic classification of components independently extracted via metafx unique algorithm and annotated for each dataset and category with Kraken2 (A) or EGGNOG v5.0 (B). Colour intensity calculated as logarithm of abundance of annotated components (blue shows high occurrence of taxa in dataset-category pair, red—low occurrence). Crohn’s disease is characterized by a loss of major intestinal taxa. Ulcerative colitis has less pronounced microbial changes.

**Figure 6:**
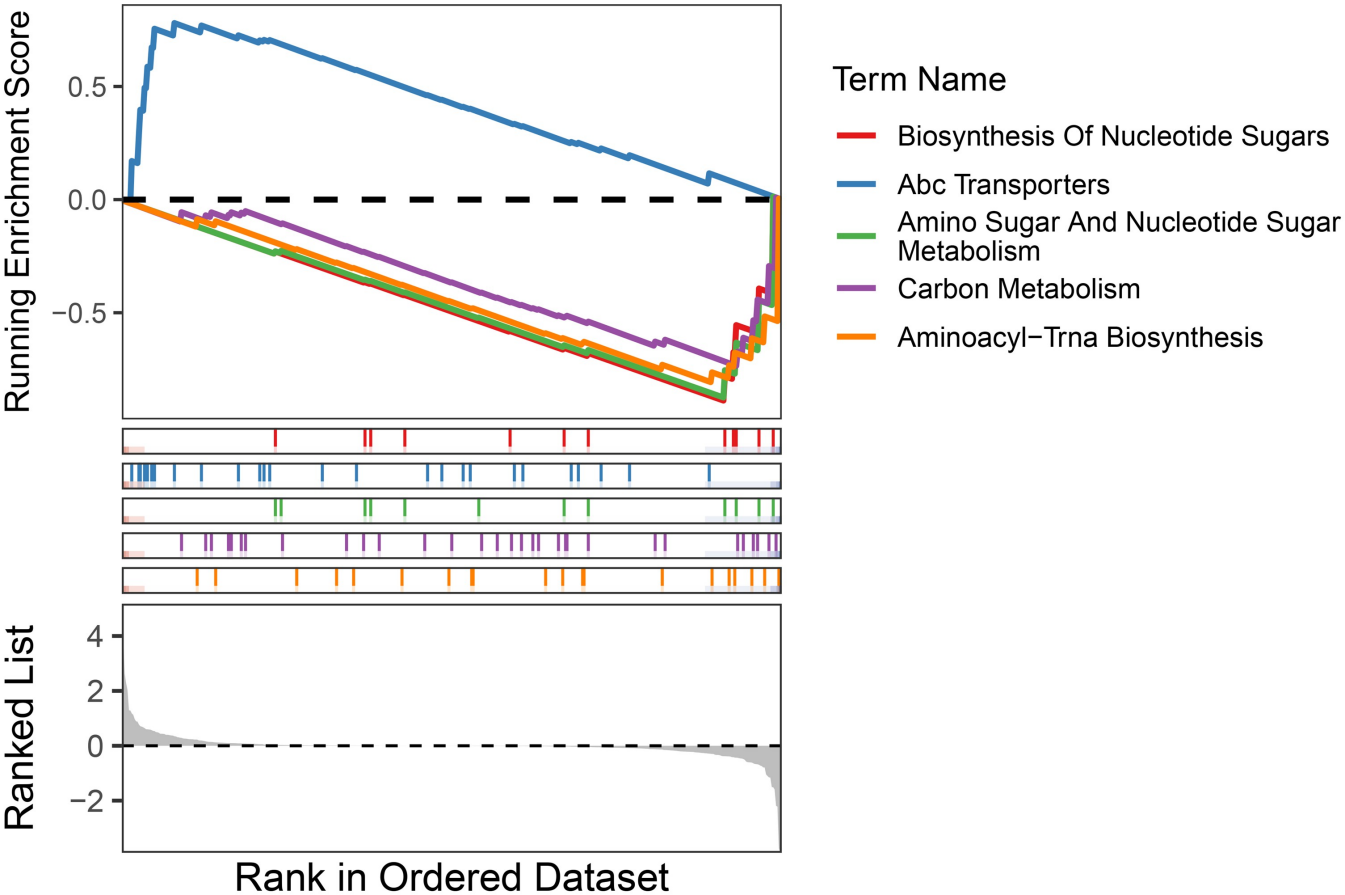
GSEA results shows that the CD group exhibited an upregulation of the ABC transporters pathway (ko02010).

We applied Random Forest classification model with 100 decision trees and other default parameters from scikit-learn library (Pedregosa *et al.* 2011). Each of four dataset was used as training, and the performance of classification model was estimated separately on other datasets. We also performed 10-fold cross validation of classifiers with the same train and test datasets. Since the feature sets for metafast method are the same in our experimental setup for all datasets, cross-validation results for this method are not absolutely fair. For CD versus UC problem, He at al. dataset was not used for training, as it lacks UC samples. The accuracy of classifications are shown on Fig. 4. Additionally to accuracy, we calculated F1-score and Matthews correlation coefficient metrics (Figs 5 and 6, available as supplementary data at *Bioinformatics* online). The overall results and conclusions remain the same, despite the little differences in best methods for several train-test pairs.

For each type of classification problem (Fig. 4A–F, G–L, and M–R) we selected the best feature extraction method for each pair of train-test datasets. In 3-class problem unique and metafast methods performed well, with metafast performing better between studies so being more generalizable. Taxonomic annotation outperformed other methods only once with Lo Sasso training dataset and He testing dataset. The increase in accuracy for the best method compared with taxonomic annotation ranges from 6% to 36 % for the same train-test pair, and from 1% to 20 % for different datasets. This fact can be explained by microbiome differences between cohorts characterized by lifestyle, dietary habits, etc. Additionally, we compared our results on Franzosa *et al.* dataset with the classification accuracy from the original article. In cross-validation study, we reached 82% accuracy by MetaFX unique module, while original article got only 65% accuracy on taxonomic species.

In 2-class IBD versus non-IBD problem, the trend remains the same. For cross-validation unique algorithms shows the best results. However, stats feature extraction method perform better for different train-test pairs. Compared with 3-class problem, the increase in classification accuracy here is less pronounced due to a simpler task formulation. In 2-class CD versus UC problem, we also observe the increase in classification accuracy obtained with MetaFX features compared with taxonomic annotation.

Unfortunately, we cannot determine the best feature extraction method to serve all experimental setups and cohorts. Nevertheless, the proposed algorithms retrieve the information hidden in sequencing data and unavailable for classical reference-based tools. The combination of methods implemented in MetaFX toolkit can provide more accurate classification results and new insights based on unannotated features.

Finally, we searched the extracted components for relevant biological explanation of the obtained results. Components from metafx unique method, that separated the experimental groups across the datasets, were annotated using Kraken2 v2.1.3 with the bacterial database built in December 2024 (Lu *et al.* 2022), and using Emapper v2.1.13 with the EggNOG v5.0 database (Huerta-Cepas *et al.* 2019, Cantalapiedra *et al.* 2021). Visualization of the taxonomic annotation results was performed using the pheatmap package v1.0.13 in R (Kolde 2019). Differential abundant taxonomic and functional features were identified using the Maaslin2 R package v1.22.0 (Mallick *et al.* 2021). Subsequently, gene set enrichment analysis (GSEA) was performed on the KEGG pathways using the gseKEGG function from the clusterProfiler package v4.16.0 (Xu *et al.* 2024), based on the Maaslin2 results. Feature ranking was performed by multiplying the Maaslin2 regression coefficients by $-{\text{log}}_{10}(\mathrm{q-value})$. Visualization of GSEA results was performed using GseaVis package v0.1.1 (Zhang *et al.* 2025).

Taxonomic profiling results for Kraken2 and EggNOG are summarized in Tables 2 and 3, available as supplementary data at *Bioinformatics* online. The top 40 differentially abundant taxonomic features are visualized in heatmaps (Fig. 5). According to the Maaslin2 differential abundance analysis (Tables 4 and 5, available as supplementary data at *Bioinformatics* online), CD group was characterized by a loss of major intestinal taxa, including key butyrate producers such as *Faecalibacterium*, *Roseburia*, *Butyrivibrio*, and *Blautia*, among others. Concurrently, we observed an increase in opportunistic bacteria from the genera *Enterocloster* and *Lachnoclostridium*. The UC group was characterized by less pronounced microbial shifts, which primarily manifested as a decrease in unclassified *Lachnospiraceae* taxa.

Results of functional annotation using the EggNOG database are summarized in Table 6, available as supplementary data at *Bioinformatics* online, alongside with the Maaslin2 differential abundance analysis results presented in Tables 7 and 8, available as supplementary data at *Bioinformatics* online. Functional analysis revealed that the CD group exhibited an upregulation of the ABC transporters pathway (ko02010) alongside a downregulation of several metabolic pathways (Fig. 6 and Table 9, available as supplementary data at *Bioinformatics* online). These included the biosynthesis of nucleotide sugars (ko01250), aminoacyl-tRNA biosynthesis (ko00970), amino sugar and nucleotide sugar metabolism (ko00520), and carbon metabolism (ko01200). Notably, the Crohn’s disease group exhibited an increase in specific orthologs for ABC transporters of phosphate (pstB: K02036, pstC: K02037), iron (afuA: K02012, afuB: K02011), and ribose (rbsA: K10441, rbsC: K10440).

Therefore, the developed MetaFX method helps to find nucleotide sequences associated with specific bacterial species and metabolic pathways highly correlated to the illness.

## 4 Discussion

Metagenomic data is intensively examined for solving various problems of fundamental and applied science. However, existing methods analysing this type of data fail to capture critical information. Reference-based methods are limited by the information in the databases leading to the streetlight effect. In contrast, reference-free methods allow deeper investigation of all available data. However, assembly-based methods require a lot of resources and k-mer-based approaches do not provide the biological interpretation of the results. Thus, there is a need to develop alternative techniques and approaches to improve the obtained results.

To overcome these limitations, MetaFX, an open-source library for feature extraction from whole-genome metagenome sequencing data and classification of sample groups have been developed. MetaFX enables rapid extraction of features in the form of DNA sequences suitable for further study and generation of biological hypotheses. MetaFX can generate a limited number of features that are the best for samples classification, which is an added advantage of the proposed approach. Features can be extracted with or without samples metadata for supervised/unsupervised analysis. Additionally, MetaFX output can be used for classification models training.

The proposed approach has significant advantages because it eliminates the step of obtaining taxonomic or functional or other features to build models, and also preserves more useful information that can be used for prediction. Furthermore, tool aims to extract the most suitable information, including “dark matter,” which can be further annotated. It should be noted that MetaFX does not fully solve the problem of cross-cohort forecasting, alike many other classification methods. In other words, a classifier trained on one dataset does not always predict samples from another dataset well. The results are highly dependent on the similarity of samples between cohorts.

The usefulness, benefits, and applicability of MetaFX were demonstrated in classification models for 590 metagenomes of patients with IBD. IBD is still a serious and highly common group of diseases that lead to destructive changes in the digestive tract. All gastrointestinal tract processes, including inflammatory disorders, are directly influenced by the human gut microbiota. However, a thorough understanding of the precise mechanisms by which bacteria are involved in this process is still lacking.

Inflammatory bowel disease involves profound gut microbial ecosystem alterations, particularly evident in CD. Our MetaFX-based analysis reveals CD is characterized by substantial depletion of keystone commensal taxa, including crucial butyrate producers such as *Faecalibacterium*, *Roseburia*, *Butyrivibrio*, and *Blautia* (Caparrós *et al.* 2021), alongside concurrent expansion of opportunistic pathogens including *Enterocloster* (Jacobsen *et al.* 2025) and *Lachnoclostridium* (Alsulaiman *et al.* 2023). Ulcerative colitis demonstrates milder dysbiosis, primarily showing reduction in unclassified *Lachnospiraceae* (Schirmer *et al.* 2019). Functional profiling reveals CD-specific metabolic reprogramming with upregulation of high-affinity ABC transporters for phosphate (pstB/C), iron (afuA/B), and ribose (rbsA/C), indicating sophisticated microbial adaptation to nutrient limitation and host nutritional immunity (Sit *et al.* 2015, Sinha *et al.* 2020), alongside concurrent downregulation of core metabolic pathways including nucleotide sugar biosynthesis, aminoacyl-tRNA biosynthesis, and carbon metabolism, reflecting global functional impairment (Schirmer *et al.* 2019, Caparrós *et al.* 2021). The perfect congruence between taxonomic and functional findings paints a coherent picture of a metabolically compromised community in CD that has lost beneficial functional capabilities while enhancing survival strategies. These results provide mechanistic insights into IBD pathophysiology and highlight the importance of targeting microbial functional restoration rather than mere compositional changes in future therapeutic strategies.

## 5 Conclusion

Microbial communities often play crucial role in host environment and comparative metagenomics can shed light on the distinct microbiome properties. We have developed the MetaFX library to automate and simplify the process of extracting meaningful features from metagenomic datasets and training classification models for new samples analysis. MetaFX offers potential users a convenient and efficient reference-free workflow for analysing metagenomic sequencing data. MetaFX is capable of processing hundreds of samples within several hours and produces ready to annotation features as fasta contigs that can be used both for bidimentional visualization and for building predictive machine learning models. Moreover, the results can be explored visually by users thanks to the accompanying software BandageNG.

Applying additional taxonomic or functional annotation methods to MetaFX features results in generation of biological hypotheses based on data analysis of large numbers of metagenomic samples. Its possible future applications may include detection of disease-associated bacteria or functional parts of human gut microbiome in disease versus control studies, tracking the effect of certain medications or interventions on gut microbiota composition, and even screening and early diagnostics of possible illnesses based on pre-trained predictive models. Finally, MetaFX analysis is not limited to human gut and can be applied to other environments such as water, soil and cow rumen.

## 6 Availability of source code and requirements

MetaFX source code is freely available on GitHub under the MIT license at github.com/ctlab/metafx. Alternatively, MetaFX can be obtained via anaconda.org/bioconda/metafx or doi.org/10.5281/zenodo.16949369. Detailed documentation is available at wiki page: github.com/ctlab/metafx/wiki. The tutorial for MetaFX based on mock microbial community analysis is available at github.com/ctlab/metafx/wiki/MetaFX-tutorial. Also a video with installation steps and basic instructions is available on YouTube: youtu.be/mTuP1jm_OlI.

- Project name: MetaFX
- Project home page: https://github.com/ctlab/metafx
- Operating system(s): Linux/macOS
- Programming language: Java, Python
- Other requirements: JRE 1.8 or higher, Python 3.9.5 or higher, other requirements are available within the documentation
- License: MIT

## Supplementary Material

btag018_Supplementary_Data

## Data Availability

All metagenomics data used in the current study were obtain freely from open databases. Details about samples and raw data can be found in the corresponding articles. Metagenomic sequences for Franzosa *et al.* (2019) are available from SRA BioProject PRJNA400072. Metagenomic sequences for Lloyd-Price *et al.* (2019) are available from SRA BioProject PRJNA398089. Metagenomic sequences for Lo Sasso *et al.* (2021) are available from the authors by the request. Metagenomic sequences for He *et al.* (2017) are available from the EBI database under the BioProject number PRJEB15371.
