# Supplementary material for: MetaFX: feature extraction from whole-genome metagenomic sequencing data
Source: Bioinformatics. 2026 Jan 20;42(2):btag018. doi: 10.1093/bioinformatics/btag018 (PMC12891910; doi:10.1093/bioinformatics/btag018)
Supplement: btag018_Supplementary_Data [file btag018_supplementary_data.zip › SFigure5.pdf]

F1-score (rows: train dataset; columns: test dataset)

3-class

train dataset	A: Taxonomy Kraken2				B: Taxonomy sylph				C: jellyfish 11-mers				D: MetaFX *metafast*				E: MetaFX *unique*				F: MetaFX *stats*			
	Franzosa	Lloyd-Price	Lo Sasso	He	Franzosa	Lloyd-Price	Lo Sasso	He	Franzosa	Lloyd-Price	Lo Sasso	He	Franzosa	Lloyd-Price	Lo Sasso	He	Franzosa	Lloyd-Price	Lo Sasso	He	Franzosa	Lloyd-Price	Lo Sasso	He
Franzosa	0,69	0,28	0,39	0,33	0,64	**0,40**	0,49	0,42	0,63	0,34	0,49	**0,46**	0,67	0,31	**0,60**	0,37	**0,83**	0,37	0,54	0,36	0,74	0,28	0,46	0,36
Lloyd-Price	0,41	0,28	0,44	0,51	0,51	0,33	0,49	0,34	0,34	0,38	0,42	0,43	**0,60**	0,33	**0,51**	0,52	0,54	**0,79**	0,48	**0,55**	0,55	0,64	0,50	0,46
Lo Sasso	0,56	**0,44**	0,62	**0,52**	0,52	0,31	0,62	0,31	0,35	0,33	0,54	0,29	**0,59**	0,43	0,62	0,46	0,34	0,26	**0,86**	0,23	0,32	0,26	0,79	0,23
He	0,41	0,30	0,37	0,90	0,42	0,36	0,34	0,94	0,32	0,25	0,31	0,83	0,41	**0,37**	0,34	**0,97**	0,37	0,35	0,33	**0,97**	**0,43**	**0,37**	**0,39**	0,92

IBD vs non-IBD

train dataset	G				H				I				J				K				L			
	Franzosa	Lloyd-Price	Lo Sasso	He	Franzosa	Lloyd-Price	Lo Sasso	He	Franzosa	Lloyd-Price	Lo Sasso	He	Franzosa	Lloyd-Price	Lo Sasso	He	Franzosa	Lloyd-Price	Lo Sasso	He	Franzosa	Lloyd-Price	Lo Sasso	He
Franzosa	0,79	0,48	0,61	0,59	0,84	0,44	**0,80**	0,45	0,71	0,52	0,74	**0,72**	0,78	0,44	0,79	0,45	**0,94**	**0,53**	0,79	0,58	0,75	0,44	0,72	0,60
Lloyd-Price	0,54	0,51	0,54	0,63	**0,78**	0,44	0,57	0,37	0,59	0,50	0,41	0,31	0,72	0,52	**0,81**	**0,78**	0,72	**0,75**	0,74	0,68	0,72	0,73	0,77	0,64
Lo Sasso	0,58	0,49	0,81	**0,67**	0,55	0,44	0,86	0,47	0,46	0,44	0,75	0,39	**0,70**	**0,54**	0,83	0,58	0,43	0,44	**1,00**	0,35	0,46	0,44	0,93	0,35
He	0,58	0,58	0,58	0,90	**0,68**	0,64	0,51	0,96	0,63	0,51	0,57	0,82	0,63	**0,66**	0,59	0,96	0,58	0,57	0,53	**0,97**	0,66	0,58	**0,64**	0,93

CD vs UC

train dataset	M				N				O				P				Q				R			
	Franzosa	Lloyd-Price	Lo Sasso	He	Franzosa	Lloyd-Price	Lo Sasso	He	Franzosa	Lloyd-Price	Lo Sasso	He	Franzosa	Lloyd-Price	Lo Sasso	He	Franzosa	Lloyd-Price	Lo Sasso	He	Franzosa	Lloyd-Price	Lo Sasso	He
Franzosa	0,80	0,47	0,46	0,36	0,73	**0,63**	0,55	**0,49**	0,75	0,57	0,54	0,47	0,71	0,58	**0,57**	0,48	0,85	0,61	0,42	0,38	**0,86**	0,48	0,48	0,48
Lloyd-Price	0,64	0,40	**0,54**	0,50	0,60	0,55	0,43	0,49	0,41	0,52	0,46	0,38	**0,70**	0,50	0,52	**0,50**	0,68	**0,81**	0,46	0,49	0,66	0,70	0,53	0,49
Lo Sasso	0,57	0,48	0,71	0,48	0,60	0,33	0,62	0,36	0,45	0,57	0,56	0,14	**0,63**	**0,58**	0,66	0,49	0,52	0,49	0,79	0,48	0,47	0,38	**0,80**	**1,00**
