# Supplementary material for: MetaFX: feature extraction from whole-genome metagenomic sequencing data
Source: Bioinformatics. 2026 Jan 20;42(2):btag018. doi: 10.1093/bioinformatics/btag018 (PMC12891910; doi:10.1093/bioinformatics/btag018)
Supplement: btag018_Supplementary_Data [file btag018_supplementary_data.zip › SFigure6.pdf]

MCC

Taxonomy Kraken2

Panel	Task	train dataset	test: Franzosa	test: Lloyd-Price	test: Lo Sasso	test: He
A	3-class	Franzosa	0,55	0,07	0,20	0,27
		Lloyd-Price	0,16	-0,03	0,17	0,56
		Lo Sasso	0,35	0,13	0,48	**0,57**
		He	0,35	0,04	0,27	0,81
G	IBD vs non-IBD	Franzosa	0,60	**0,17**	0,31	0,40
		Lloyd-Price	0,27	0,24	0,10	0,41
		Lo Sasso	0,21	0,05	0,63	**0,48**
		He	0,40	0,16	0,40	0,79
M	CD vs UC	Franzosa	0,60	0,26	0,13	0,00
		Lloyd-Price	0,31	-0,13	0,07	0,00
		Lo Sasso	0,25	0,09	**0,42**	0,00

Taxonomy sylph

Panel	Task	train dataset	test: Franzosa	test: Lloyd-Price	test: Lo Sasso	test: He
B	3-class	Franzosa	0,48	0,17	0,31	**0,41**
		Lloyd-Price	0,32	0,09	0,22	0,29
		Lo Sasso	0,26	0,02	0,46	0,10
		He	0,35	0,20	0,24	0,88
H	IBD vs non-IBD	Franzosa	0,70	0,00	0,59	0,23
		Lloyd-Price	**0,57**	-0,06	0,33	0,10
		Lo Sasso	0,31	0,00	0,73	0,25
		He	**0,50**	0,31	0,34	0,91
N	CD vs UC	Franzosa	0,46	0,26	0,10	0,00
		Lloyd-Price	0,27	0,22	-0,10	0,00
		Lo Sasso	0,21	-0,11	0,27	0,00

jellyfish 11-mers

Panel	Task	train dataset	test: Franzosa	test: Lloyd-Price	test: Lo Sasso	test: He
C	3-class	Franzosa	0,45	0,03	0,36	0,40
		Lloyd-Price	0,07	0,10	0,19	0,23
		Lo Sasso	0,06	0,12	0,35	0,22
		He	0,12	0,10	0,17	0,65
I	IBD vs non-IBD	Franzosa	0,46	0,05	0,49	**0,51**
		Lloyd-Price	0,20	0,02	0,10	0,00
		Lo Sasso	0,11	0,00	0,50	0,07
		He	0,28	0,24	0,17	0,64
O	CD vs UC	Franzosa	0,51	0,14	0,12	0,00
		Lloyd-Price	0,00	0,09	-0,07	0,00
		Lo Sasso	-0,05	0,14	0,13	0,00

MetaFX *metafast*

Panel	Task	train dataset	test: Franzosa	test: Lloyd-Price	test: Lo Sasso	test: He
D	3-class	Franzosa	0,50	0,13	0,46	0,39
		Lloyd-Price	**0,41**	0,04	**0,30**	0,60
		Lo Sasso	**0,41**	**0,14**	0,44	0,50
		He	0,36	**0,21**	0,24	**0,93**
J	IBD vs non-IBD	Franzosa	0,58	0,00	**0,61**	0,23
		Lloyd-Price	0,53	0,11	**0,65**	**0,64**
		Lo Sasso	**0,48**	**0,19**	0,65	0,39
		He	0,45	**0,34**	0,42	**0,91**
P	CD vs UC	Franzosa	0,43	0,30	**0,26**	0,00
		Lloyd-Price	**0,41**	0,13	**0,17**	0,00
		Lo Sasso	**0,27**	**0,16**	0,32	0,00

MetaFX *unique*

Panel	Task	train dataset	test: Franzosa	test: Lloyd-Price	test: Lo Sasso	test: He
E	3-class	Franzosa	**0,80**	**0,18**	**0,47**	0,27
		Lloyd-Price	0,39	**0,57**	0,29	**0,65**
		Lo Sasso	0,16	-0,03	**0,63**	0,14
		He	0,29	0,19	0,23	0,83
K	IBD vs non-IBD	Franzosa	**0,89**	0,13	0,60	0,37
		Lloyd-Price	0,51	**0,78**	0,48	0,47
		Lo Sasso	0,00	0,00	**1,00**	0,00
		He	0,40	0,20	0,36	0,83
Q	CD vs UC	Franzosa	**0,88**	**0,37**	0,05	0,00
		Lloyd-Price	0,36	**0,41**	0,08	0,00
		Lo Sasso	0,04	0,03	0,26	0,00

MetaFX *stats*

Panel	Task	train dataset	test: Franzosa	test: Lloyd-Price	test: Lo Sasso	test: He
F	3-class	Franzosa	**0,80**	0,13	0,26	0,40
		Lloyd-Price	0,37	0,39	0,28	0,39
		Lo Sasso	0,19	-0,01	**0,63**	0,00
		He	**0,37**	**0,21**	**0,31**	0,83
L	IBD vs non-IBD	Franzosa	0,65	0,00	0,49	0,42
		Lloyd-Price	0,45	0,53	0,54	0,40
		Lo Sasso	0,16	0,00	0,82	0,00
		He	0,42	0,18	**0,47**	0,83
R	CD vs UC	Franzosa	**0,88**	0,14	0,16	0,00
		Lloyd-Price	0,35	0,10	0,07	0,00
		Lo Sasso	0,26	-0,08	0,26	0,00
