# Supplementary material for: MetaFX: feature extraction from whole-genome metagenomic sequencing data
Source: Bioinformatics. 2026 Jan 20;42(2):btag018. doi: 10.1093/bioinformatics/btag018 (PMC12891910; doi:10.1093/bioinformatics/btag018)
Supplement: btag018_Supplementary_Data [file btag018_supplementary_data.zip › STable1.pdf]

Table 1. Datasets from human gut microbiome with inflammatory bowel diseases

Dataset	Total samples	Crohn's disease	Ulcerative colitis	Control	# reads (mean±SD), mln	# k-mers (mean±SD), mln	# k-mers > 1 (mean±SD), mln
Franzosa et al. [2019]	220	88	76	56	42.9 ± 33.3	490 ± 271	205 ± 105
Lloyd-Price et al. [2019]	130	65	38	27	24.3 ± 11.8	312 ± 152	129 ± 55
Lo Sasso et al. [2021]	124	40	42	42	55.3 ± 8.9	671 ± 343	256 ± 100
He et al. [2017]	116	63	0	53	55.8 ± 7.3	351 ± 123	179 ± 70
**Total**	590	256	156	178			

References

E. A. Franzosa et al. Gut microbiome structure and metabolic activity in inflammatory bowel disease. *Nature microbiology*, 4(2): 293–305, 2019.

Q. He et al. Two distinct metacommunities characterize the gut microbiota in crohn's disease patients. *Gigascience*, 6(7):gix050, 2017.

J. Lloyd-Price et al. Multi-omics of the gut microbial ecosystem in inflammatory bowel diseases. *Nature*, 569(7758):655–662, 2019.

G. Lo Sasso et al. Inflammatory bowel disease–associated changes in the gut: focus on kazan patients. *Inflammatory bowel diseases*, 27(3):418–433, 2021.
