# Supplementary material for: MetaFX: feature extraction from whole-genome metagenomic sequencing data
Source: Bioinformatics. 2026 Jan 20;42(2):btag018. doi: 10.1093/bioinformatics/btag018 (PMC12891910; doi:10.1093/bioinformatics/btag018)
Supplement: btag018_Supplementary_Data [file btag018_supplementary_data.zip › SFigure1.pdf]

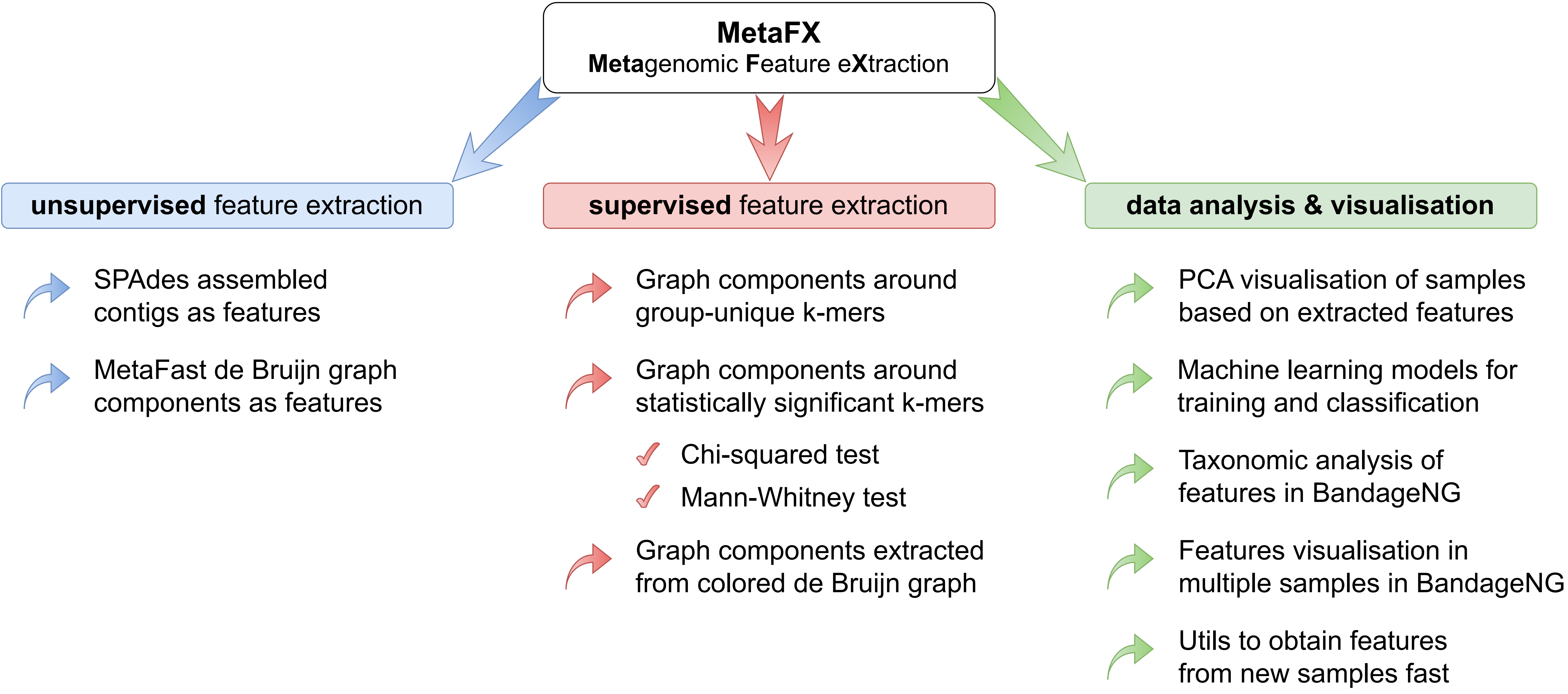
MetaFX
Metagenomic Feature eXtraction
unsupervised feature extraction
supervised feature extraction
data analysis & visualisation
SPAdes assembled contigs as features
MetaFast de Bruijn graph components as features
Graph components around group-unique k-mers
Graph components around statistically significant k-mers
Chi-squared test
Mann-Whitney test
Graph components extracted from colored de Bruijn graph
PCA visualisation of samples based on extracted features
Machine learning models for training and classification
Taxonomic analysis of features in BandageNG
Features visualisation in multiple samples in BandageNG
Utils to obtain features from new samples fast
