# Supplementary material for: MetaFX: feature extraction from whole-genome metagenomic sequencing data
Source: Bioinformatics. 2026 Jan 20;42(2):btag018. doi: 10.1093/bioinformatics/btag018 (PMC12891910; doi:10.1093/bioinformatics/btag018)
Supplement: btag018_Supplementary_Data [file btag018_supplementary_data.zip › SFigure3.pdf]

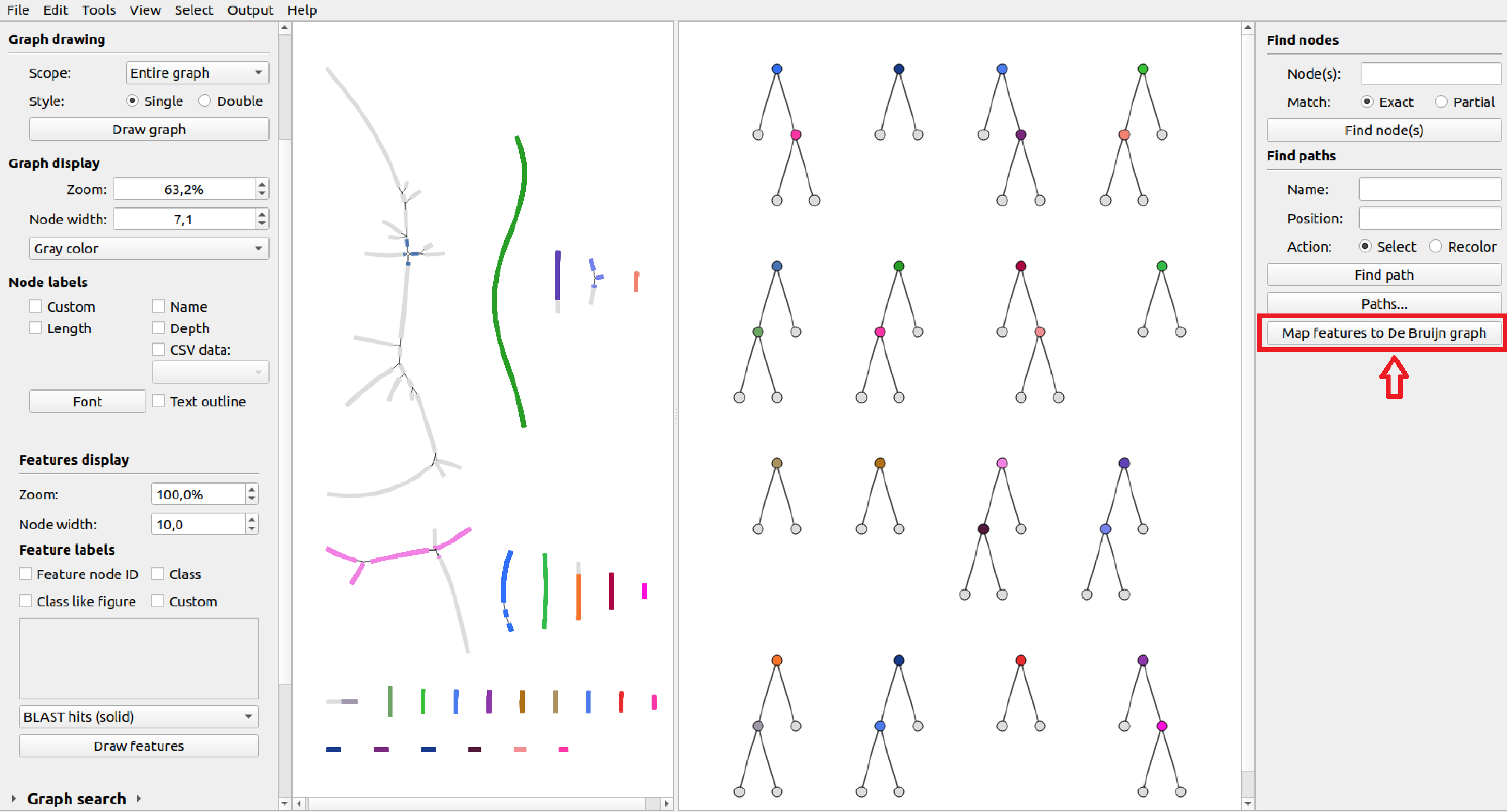
File Edit Tools View Select Output Help
Graph drawing
Scope: Entire graph
Style: Single Double
Draw graph
Graph display
Zoom: 63,2%
Node width: 7,1
Gray color
Node labels
Custom
Name
Length
Depth
CSV data:
Font
Text outline
Features display
Zoom: 100,0%
Node width: 10,0
Feature labels
Feature node ID
Class
Class like figure
Custom
BLAST hits (solid)
Draw features
Graph search
Find nodes
Node(s):
Match: Exact Partial
Find node(s)
Find paths
Name:
Position:
Action: Select Recolor
Find path
Paths...
Map features to De Bruijn graph
