# Supplementary figures and images for: MetaFX: feature extraction from whole-genome metagenomic sequencing data
Source: Bioinformatics. 2026 Jan 20;42(2):btag018. doi: 10.1093/bioinformatics/btag018 (PMC12891910; doi:10.1093/bioinformatics/btag018)

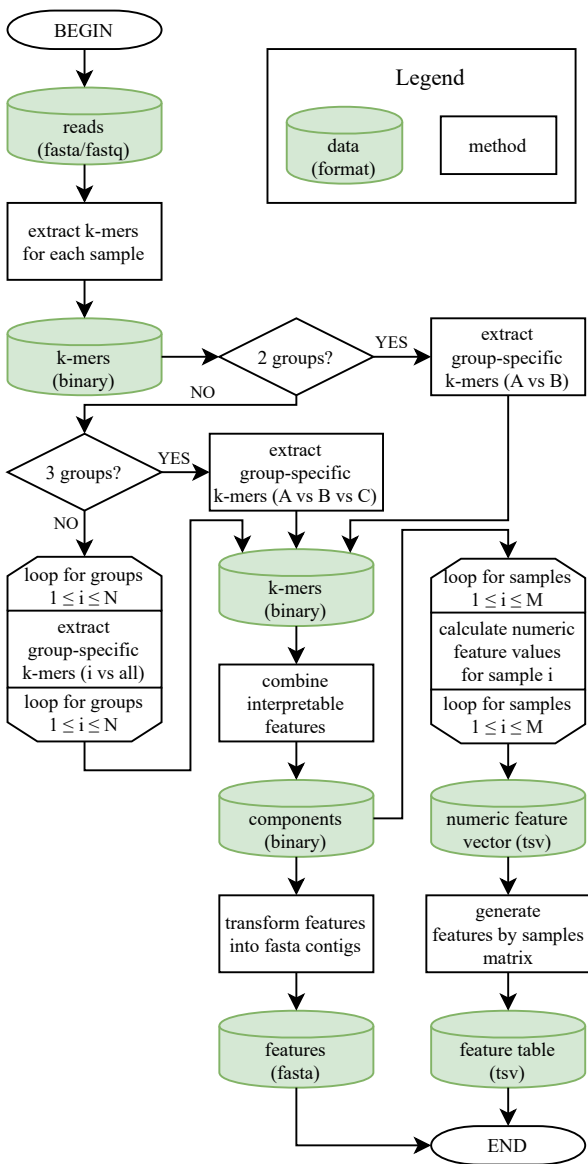

Supplement: btag018_Supplementary_Data [file btag018_supplementary_data.zip › SFigure2.pdf]

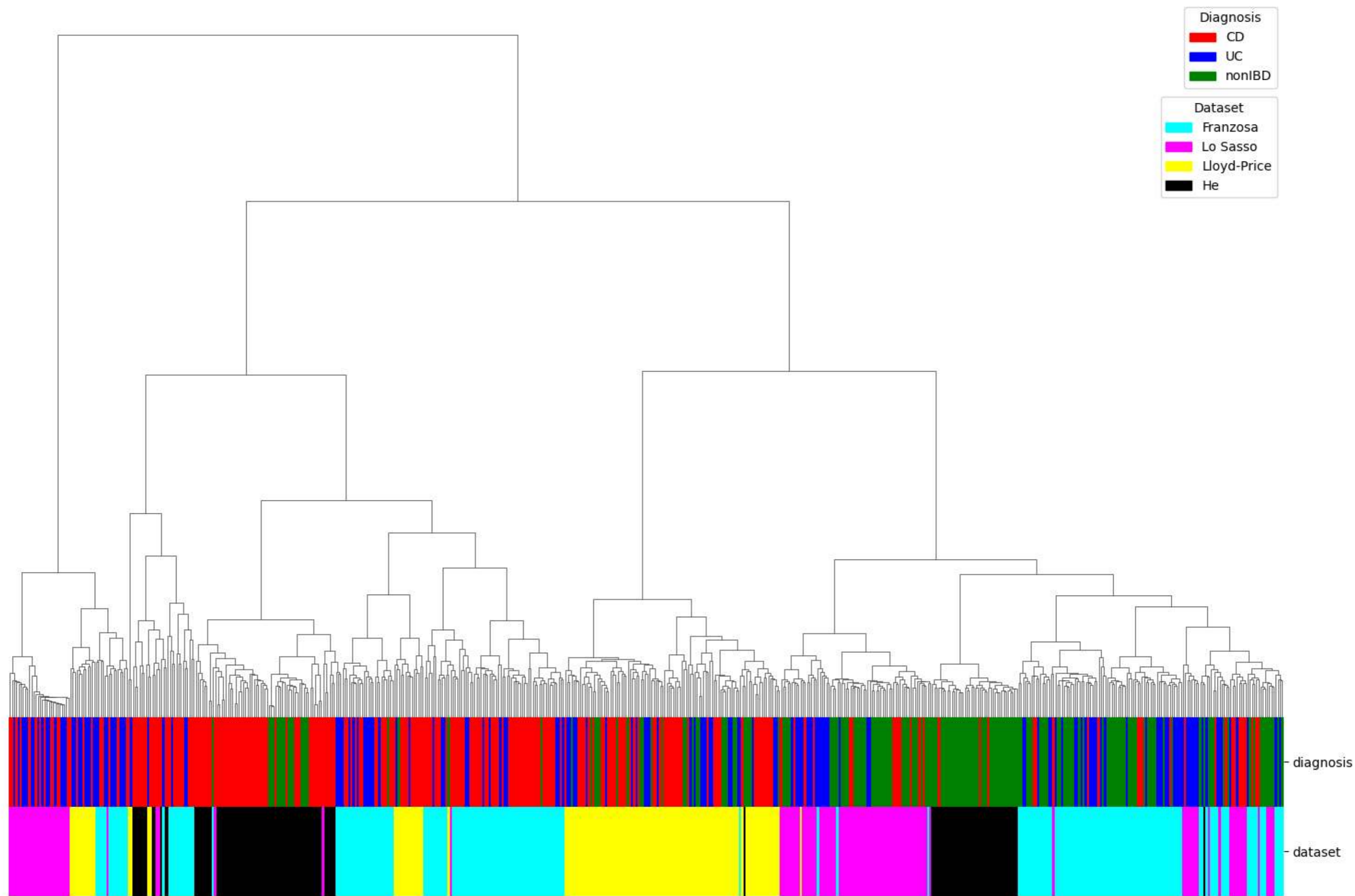

Supplement: btag018_Supplementary_Data [file btag018_supplementary_data.zip › SFigure4.pdf]
